# Characterization and Mutational Analysis of a Monogalactosyldiacylglycerol Synthase Gene *OsMGD2* in Rice

**DOI:** 10.3389/fpls.2019.00992

**Published:** 2019-08-02

**Authors:** Rasbin Basnet, Jiarun Zhang, Nazim Hussain, Qingyao Shu

**Affiliations:** ^1^National Key Laboratory of Rice Biology, Institute of Crop Sciences, Zhejiang University, Hangzhou, China; ^2^Hubei Collaborative Innovation Center for the Grain Industry, Yangtze University, Jingzhou, China; ^3^Zhejiang Key Laboratory of Crop Germplasm Resources, College of Agriculture and Biotechnology, Zhejiang University, Hangzhou, China

**Keywords:** galactolipids, MGDG, OsMGD2, endosperm, rice

## Abstract

Monogalactosyldiacylglycerol (MGDG) and digalactosyldiacylglycerol (DGDG) are the two predominant galactolipids present in the photosynthetic membrane in many photosynthetic organisms, including algae and higher plants. These galactolipids are the main constituents of thylakoid membrane and are essential for chloroplast biogenesis and photoautotrophic growth. *In silico* analysis revealed that rice (*Oryza sativa* L.) genome has three genes encoding MGDG synthase (*OsMGD1, 2*, and *3*). Although subcellular localization analysis demonstrated that OsMGD2 is localized to chloroplast, its expression was observed mainly in anther and endosperm, suggesting that MGDG might have an important role in the development of flower and grain in rice. Knock-out mutants of *OsMGD2* were generated employing the CRISPR/Cas9 system and their morphology, yield and grain quality related traits were studied. The leaf of *osmgd2* mutants showed reduced MGDG (∼11.6%) and DGDG (∼9.5%) content with chlorophyll **a** content decreased by ∼23%, consequently affecting the photosynthesis. The mutants also exhibited poor agronomic performance with plant height and panicle length decreased by ∼12.2 and ∼7.3%, respectively. Similarly, the number of filled grains per panicle was reduced by 43.8%, while the 1000 grain weight was increased by ∼6.3% in the mutants. The milled rice of mutants also had altered pasting properties and decreased linoleic acid content (∼26.6%). Put together, the present study demonstrated that *OsMGD2* is the predominantly expressed gene encoding MGDG synthase in anther and grain and plays important roles in plant growth and development, as well as in grain quality.

## Introduction

Galactolipids are a major class of higher plant glycerolipids, in which galactose is bound at the glycerol sn-3 position in O-glycosidic linkage to diacylglycerol. Monogalactosyldiacylglycerol (MGDG) and digalactosyldiacylglycerol (DGDG) are the two predominant galactolipids in photosynthetic membranes of cyanobacteria, algae and higher plants, accounting for around 50 and 20% of the chloroplast lipid, respectively (Dörmann and Benning, 2002; Kalisch et al., 2016). These galactolipids form the indispensable matrix in the thylakoid membrane of chloroplast, where photochemical and electron transport reactions of oxygenic photosynthesis occur (Mizusawa and Wada, 2012; Dörmann, 2013; Kobayashi et al., 2016). MGDG and DGDG have also been detected in extraplastidic membranes, including the plasma membrane, tonoplast, endoplasmic reticulum, and Golgi membranes, implying their non-photosynthetic functions in higher plants (Hartel et al., 2000; Wang et al., 2014). However, in non-photosynthetic tissues such as roots, the abundance of galactolipids is comparatively reduced due to less abundance of chloroplast (Li-Beisson et al., 2016).

Glycerolipids biosynthesis in higher plants occurs via two different pathways. In (a) Prokaryotic pathway, the fatty acids (FAs) synthesized in the plastid are directly used up in the plastid, whereas in (b) Eukaryotic pathway, the FAs are first exported from plastid as CoA esters to endoplasmic reticulum or other sites and then returned to plastid for the synthesis of membrane lipids (Boudière et al., 2014; Kalisch et al., 2016). MGDG is synthesized by adding a molecule of galactose onto the diacylglycerol (DAG) by the MGDG synthase. Similarly, a second galactose is then transferred onto the MGDG by DGDG synthase to synthesize DGDG (Ohlrogge and Browse, 1995; Dörmann et al., 1999). Therefore, MGDG synthase is the principal enzyme for the synthesis of both MGDG and DGDG and has been found crucial for plant growth and development (Dörmann and Benning, 2002; Kalisch et al., 2016).

In *Arabidopsis*, the MGDG synthase is encoded by two types of genes, namely type-A (*AtMGD1*) and type-B (*AtMGD2* and *AtMGD3*) (Awai et al., 2001; Kobayashi et al., 2004). AtMGD1 is essential to synthesize the bulk of MGDG in leaf, and the knock-out mutant of *AtMGD1*(*mgd1-2*) had a ∼98% reduction in the amount of MGDG (Kobayashi et al., 2007). In another study, the defective *AtMGD1* mutant (*mgd1-1*), carrying a T-DNA insertion in the *MGD1* promoter region, had ∼50% reduction in total chlorophyll content and 42% depletion in MGDG content in its leaves (Jarvis et al., 2000). *AtMGD1* was reported to be indispensable for the synthesis of MGDG in the inner envelope of chloroplast and its complete loss of function resulted in impairment of photosynthetic ability and photoautotrophic growth (Kobayashi et al., 2007). MGDG has also been found crucial to confer tolerance to various environmental stresses, including phosphorous deficiency (Kobayashi et al., 2004, 2009), salt stress, submergence, and wounding in plants (Qi et al., 2004; Klecker et al., 2014). Tobacco plants overexpressing *AtMGD1* had improved salt tolerance, higher chlorophyll levels and significantly higher MGDG and DGDG content in leaves (Wang et al., 2014). *AtMGD2* and *AtMGD3* have also been found to be highly active under phosphate limited conditions in photosynthetic as well as non-photosynthetic tissues, particularly in roots (Awai et al., 2001; Benning and Ohta, 2005; Kobayashi et al., 2009). In maize, the complete loss of MGD1 function caused failure in the development of both endosperm and embryo, and led to kernel lethality in a null mutant (Myers et al., 2011), implying the importance of MGDG in non-photosynthetic tissues.

MGDG synthases have been studied intensively in the photosynthetic tissues of many green plants, mostly in *Arabidopsis* as well as in non-photosynthetic tissues under various stresses. However, the identification and characterization of all MGDG synthase genes in rice has not been reported well yet, although one of the MGDG synthase gene has been previously cloned and shown to be overexpressed during submergence, drought and salinity conditions, as well as ethephon and gibberellin treatments (Qi et al., 2004). In this study, we identified three *AtMGD* homolog genes in rice (*OsMGD1, 2, 3*) and studied their genomic structures, conserved motifs and domains, and phylogenetic relationship with the MGDG synthase genes in *Arabidopsis* and maize. *OsMGD2* was found to be highly expressed in anther and endosperm, suggesting a non-photosynthetic function of MGDG in the development of flower and grain in rice. Further, we employed the reverse genetic approach, using the CRISPR/Cas9 system, to generate mutants of *OsMGD2* and elucidated its role in photosynthesis, seed quality and rice productivity.

## Materials and Methods

### *In silico* Analysis: Identification and Analysis of MGDG Synthase Genes

Multiple databases such as Gramene^1^, KEGG (Kyoto Encyclopedia of Genes and Genomes^2^), PMN (Plant Metabolic Network^3^) were searched for the identification of genes encoding MGDG synthase through investigation of pathways for MGDG synthesis in rice. The protein sequences of the *Arabidopsis* MGDG synthase encoding genes (*AtMGD1, 2 and 3*) obtained from the TAIR database^4^ were used as queries to perform BLAST against the rice and maize genome in NCBI (National Center for Biotechnology Information^5^) and Gramene databases to confirm the genes and check for any redundancy. The positional information, predicted protein length, molecular weight and isoelectric point of rice MGDG synthases were obtained from RGAP (Rice Genome Annotation Project^6^) and Gramene databases. The GSDS (Gene Structure Display server^7^) was used for gene structure (exon-intron distribution) analysis. EBI’s HMMER^8^ was used for domain analysis to identify and locate the Pfam domain region. MEGA 7.0 was used to perform multiple sequence alignment using CLUSTALW and the maximum likelihood (ML) in the Jones-Taylor-Thornton (JTT) model was used to create the phylogenetic tree. The conserved motifs of MGDG synthases were analyzed using the MEME 4.12 with number of different motifs as 20 and default parameters of minimum motif width as 6 and a maximum motif width set to 50^9^. The tissue specific expression of genes encoding MGDG synthase in rice were obtained from the RGAP database, while gene expression data for *Arabidopsis* and maize were obtained from the BAR (The Bio-Analytic Resource for Plant Biology) database^10^ for comparative analysis.

### Laboratory Experiment

#### Generation of *osmgd2* Mutants Using CRISPR/Cas9

**FIGURE 1:**
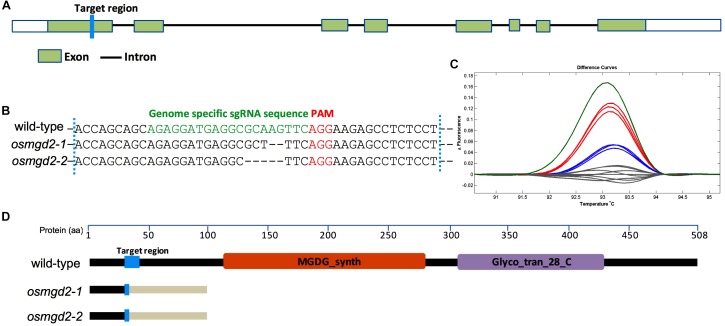
Targeted mutagenesis of *OsMGD2* using CRISPR/Cas9 system. **(A)** Gene structure of *OsMGD2* with the target region shown in blue. **(B)** DNA sequences of the target region with PAM and base pairing sequence of sgRNA are shown in red and green, respectively. *osmgd2-1* and *osmgd2-2* have 2-bp and 5-bp deletion, respectively. **(C)** High resolution melting (HRM) analysis of target fragments in wild-type (gray) and mutants (colored). **(D)** Schematic representation of truncation in protein translation in the mutants. MGDG_synth and Glyco_tran_28_C are two domains shown in brown and purple color. Changes in amino acid sequences in the mutants are shown by pale white color after the target region.

The CRISPR vector pHUN4c12 (Xu et al., 2014) was used to construct genome editing vectors for the development of *OsMGD2* (LOC_Os02g55910/ Os02g0802700) mutants. CRISPR-P 1.0^11^ was used to design the base pairing sequence (M2-T/-B) of the sgRNA targeting the first exon (Figure 1A,B) and the top and bottom oligos were annealed together using Annealing buffer 5X (Beyotime). Briefly, pHUN4c12 was digested using the BsaI-HF restriction enzyme (NEB) and purified using the Axygen^®^ DNA gel purification kit (Capitol Scientific, TX, United States). The linearized vector and the annealed oligos were ligated using the T4 DNA ligase. The ligated vector was transformed into chemically competent DH5α using the heat shock treatment and insertion of the base pairing sequence was verified by sequencing using the primer (Seq-F). The construct was introduced into the chemically competent *Agrobacterium* (EHA105) and transformed into rice callus induced from a *japonica* rice cultivar Xidao #1 according to Liu et al. (2019).

#### Identification and Growth of Mutants

DNA was extracted from the leaf sample of transgenic T_0_ plants using the CTAB method. High resolution melting (HRM) analysis was performed (Li et al., 2018) to check for mutation in the targeted fragment using the primer pairs M2-F and M2-R. The target fragments with different colored curves on HRM analysis (Figure 1C) were selected for sequencing and UNIPRO UNIGENE software was used to study the nature of mutation. Two mutants with different mutations were then selected for further growth and the ExPASy translate tool^12^ was used to study the truncation in the protein in the mutants. Transgene-free T_1_ plants were screened by PCR of hygromycin resistance gene using the primer pair Hyg-F and Hyg-R. Agronomic traits were studied by growing the T_3_ mutants in three replication plots of 6 × 8 plants with 20 cm spaces between the plants. Agronomic data were collected at the grain maturity stage (40 DAF). Initially, the average number of tillers was calculated in each plot. The plant with average tiller number were selected to record plant height, panicle length and grains per panicle. 1000 grain weight was calculated by taking three replicates from each plot and plot weight was calculated as 48 plants per plot.

#### Measurement of Chlorophyll and Photosynthetic Parameters

Flag leaves were collected at the day of flowering and chlorophyll content was quantified according to Lichtenthaler and Wellburn (1983), Spectrophotometer used: Shimadzu, UV-2550, Japan Excitation wavelength: 470 nm, 649 nm, 665 nm using 96% (v/v) ethanol. Similarly, the photosynthetic rate, stomatal conductance and transpiration rate were analyzed using the portable photosynthetic system (LiCOR LI-6400XT).

#### Lipid Composition Analysis

Fatty Acids content in milled rice was analyzed according to Zhu et al. (2012) taking 0.5 g of rice flour for lipid extraction in a 8 mL of extraction solution (chloroform/isopropanol, 2:1 v/v). The supernatant (5 mL) was transferred to a new 10 mL glass tube and dried under N_2_ and 500 mL of extraction solvent was added to re-dissolve the dried lipid extract. The FAMEs (fatty acid methyl esters) were prepared by adding 1 mL of 1% MeOH/H_2_SO_4_ (v/v), capped tightly and kept in a water bath at 80°C for 1 h. After the tubes were cooled to room temperature, 1 mL of 0.9% NaCl was added followed by 1 mL of hexane, vortexed briefly and centrifuged at 2500 rpm for 3 min. The supernatant containing the FAMEs (1 mL) was transferred into GC vials and analyzed using GC-FID (Li et al., 2013). 30 mg of leaf discs (from flag leaf) were also used for lipid extraction, lipid classes were separated on a TLC plate, and visualized using iodine staining (Wang and Benning, 2011). The MGDG and DGDG spots were scraped using a razor blade and FAME was prepared as mentioned above.

#### Rapid Viscosity Analysis

Rice viscosity analysis using a rapid viscosity analyzer (RVA, Model 3D; Newport Scientific) was performed to analyze the difference in pasting properties of the milled rice flour. Three grams of white rice flour was put in an aluminum container and 25 mL of water was added. The canister was then loaded and analyzed according to the method of Tong et al. (2014). The viscosity was measured in Rapid Visco Units (RVU).

#### Gene Expression Analysis

Total RNA was extracted from flag leaf and anther samples collected on the day of flowering using the RNAprep pure kit (Tiangen Biotech, Beijing, China) and reverse-transcribed using Primescript RT reagent kit (Takara, Japan). qRT-PCR was performed on Roche Illuminator (Penzberg, Germany)using Hieff^TM^ qPCR SYBR^®^ master mix (Yeasen, China) with four biological replicates each with two technical repeats. *OsActin* was used as the internal reference and the relative expression levels were measured using the 2^-ΔΔCt^ analysis method. The list of primers and oligos used in the study are listed out in Supplementary Table S1.

#### Protein Subcellular Localization of OsMGD2

cDNA of *OsMGD2* was PCR-amplified (Primer: M2-cD-F/-R) using KOD Neo plus polymerase (TOYOBO, Japan) and initially inserted into pMD18-T vector for sequence confirmation. The cDNA was further PCR amplified from the recombinant pMD18-T-vector using primer pair M2-HR-F/-R and inserted into the pGFP-EGFP vector via XhoI and NcoI restriction sites using the homologous recombination method (Vazyme Biotech co., Ltd.). Protoplasts were extracted from 10 days old rice seedlings and the recombinant pGFP-EGFP construct with *OsMGD2* cDNA was transfected into the extracted protoplasts using polyethylene glycol (He et al., 2016), incubated for 14 h. Rice protoplasts were imaged at room temperature using a LSM780 inverted confocal microscope with an Argon laser (Germany). GFP was excited at 488 nm, and the emitted light was captured at 500–550 nm. Chlorophyll autofluorescence was excited at 488 nm, and the emitted light was captured at 650–750 nm.

## Results

### Identification and Characterization of MGDG Synthase Genes in Rice

#### MGDG Synthase in Rice Is Encoded by Three Genes

Three homologous genes encoding MGDG synthase were identified in rice genome. Based on their phylogenetic relationship with the *Arabidopsis* MGDG synthase genes, they are assigned names as *OsMGD1, OsMGD2*, and *OsMGD3* (Supplementary Figure S1 and Supplementary Table S2). These genes are located in different chromosomes and encode proteins of different length, molecular weight and isoelectric points (Supplementary Table S2). Gene structure study shows variations in the intron-exon length among *Arabidopsis*, rice and maize. Eight exons are present in most of the genes except *AtMGD2, ZmMGD2* and *ZmMGD3* which has only 6, 6 and 5 exons, respectively (Supplementary Figure S1A). Phylogenetic analysis indicates that *AtMGD1, OsMGD1*, and *ZmMGD1* belong to the type-A group, while *AtMGD2 AtMGD3, OsMGD2, OsMGD3, ZmMGD2*, and *ZmMGD3* belonging to the type-B group form a separate clade (Supplementary Figure S1B).

Ten motifs are conserved among all *Arabidopsis*, rice and maize MGDG synthase proteins, while 11 motifs are shared among AtMGD1, OsMGD1 and ZmMGD1 (Supplementary Figure S1C). The N-terminal end of AtMGD1, OsMGD2 and ZmMGD2 also share a common motif (No. 12). All of the MGDG synthase proteins in *Arabidopsis*, rice and maize have two domains, MGDG_synth (PF06925) and Glyco_tran_28_C (PF04101). MGDG_synth spans across motifs 9, 1, 5, 3, and 8, whereas Glyco_tran_28_C across motifs 4, 6, 2, and 10.

RNA-seq FPKM expression values obtained from the RGAP database showed that expression of *OsMGD1* was the highest in leaves, followed by *OsMGD2* and *OsMGD3*. However, in endosperm and anther, *OsMGD2* was found to be the only highly expressed MGDG synthase encoding gene (Supplementary Figure S2). *OsMGD1* was also found to be expressed in floral tissues and seed but the expression was relatively low. The expression of *OsMGD3* was found negligible in all tissues.

#### OsMGD2 Is Localized in the Chloroplast

**FIGURE 2:**
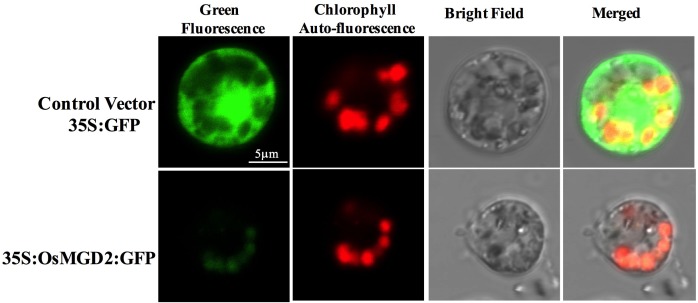
Transient expression of 35S:OsMGD2:GFP fusion in rice protoplast. Protoplasts transfected with control vector (35S:GFP) have bright GFP signal distributed throughout the cell, while those with 35S:OsMGD2:GFP have fluorescent signal (in green) localized in the chloroplast, confirmed by the auto-fluorescent signal (in red) from chlorophyll.

Protein subcellular localization study showed green fluorescent signal from GFP fused proteins transiently expressed in the protoplast of rice. The chloroplasts were visualized by the auto fluorescence of chlorophyll and appeared in red at the periphery of the protoplast. The control vector (35S:GFP) has bright GFP signal (in green) distributed throughout the cell, while the fluorescence from 35S:OsMGD2:GFP fused proteins were only localized in the chloroplast (Figure 2), suggesting the localization of OsMGD2 in chloroplast.

### Generation of Mutants and Their Effects on Seed Quality

#### Generation of Two *osmgd2* Mutant Lines

The CRISPR/Cas9 based target mutagenesis of the first exon of *OsMGD2* resulted in two different types of mutation, a 2-bp deletion and a 5-bp deletion, which were 3-bp away from the PAM (Figure 1B). Analysis of translated amino acid sequences of the mutants revealed that the protein translation is altered at the mutation site and terminated very shortly in the two mutants (Figure 1D).

**FIGURE 3:**
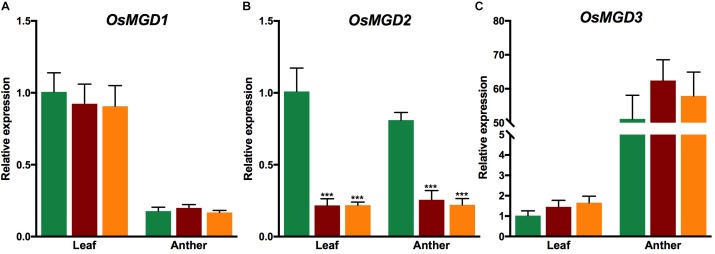
Relative expression of *OsMGD1* **(A)**, *OsMGD2* **(B)** and *OsMGD3* **(C)** in leaf and anther of Xidao #1 and its two mutants *osmgd2-1* and *osmgd2-2*. The expression levels were first normalized to the internal control gene *OsACTIN* and reported relative to wild-type leaf (assigned a value of 1). All values represent means ± standard deviations of four biological repeats. Asterisk represents statistically significant differences between wild-type and mutants (Tukey’s test, ^∗^*P* < 0.05, ^∗∗^*P* < 0.01, ^∗∗∗^*P* < 0.001).

qRT-PCR showed that the expression of *OsMGD2* was significantly reduced in both *osmgd2* mutants in leaf and anther; however, the mutations did not have any significant effect on the expression of other *OsMGD* genes in both leaf and anther (Figure 3).

#### Mutants Have Reduced Lipid Content in Leaf and Milled Rice

**FIGURE 4:**
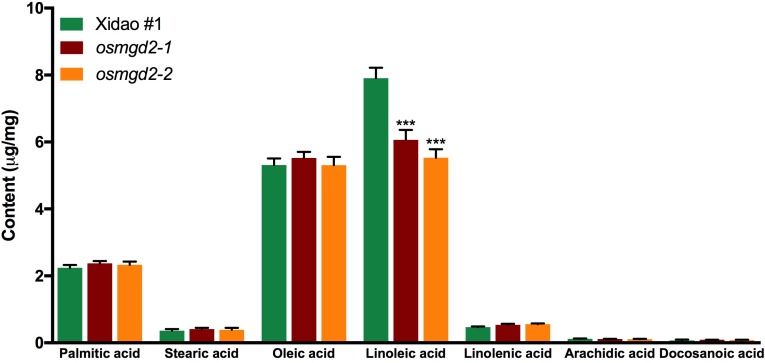
Fatty acid content and composition of milled rice of wild-type cultivar Xidao #1 and its two mutants *osmgd2-1* and *osmgd2-2.* All values represent ± standard deviations of six biological repeats. Asterisk represents statistically significant differences between wild-type and mutants (Tukey’s test, ^∗∗∗^*P* < 0.001).

Fatty acid composition analysis showed linoleic acid (18:2) was the predominant fatty acid, constituting 47.9% of the total fatty acids in wild-type milled rice (Figure 4). The amount of oleic acid and palmitic acid were also substantial, amounting to 32.2% and 13.5% of the total fatty acid content, while the amount of stearic acid, linolenic acid, arachidic acid and docosanoic acid were minimal. In the mutants, the total fatty acid content was significantly reduced to 15.4 and 14.3 mg/g in the two mutants from 16.5 mg/g in the wild-type. Among the fatty acids, only linoleic acid was significantly lowered in mutant rice while changes of other fatty acids content were insignificant (Figure 4).

**FIGURE 5:**
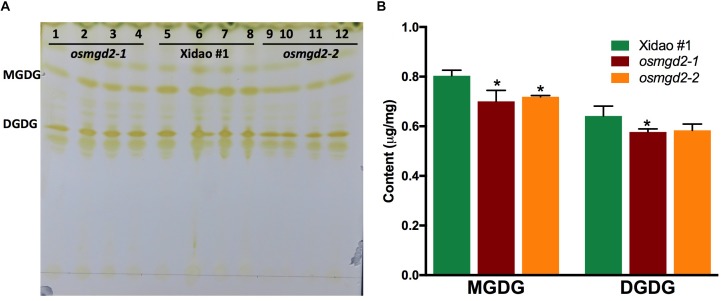
Separation and measurement of galactolipids extracted from leaves. **(A)** Separation of lipids on Thin Layer Chromatography (TLC) plate and visualized irreversibly by staining with iodine vapor. Lanes: 1–4 *osmgd2-1*, lane:5–8 Xidao #1 and lanes: 9–12 *osmgd2-2*. **(B)** Total fatty acid content in MGDG and DGDG (from 5A) in leaf of wild-type cultivar Xidao #1 and its two mutants *osmgd2-1* and *osmgd2-2*. All values represent means ± standard deviations of three biological repeats. Asterisk represents statistically significant differences between wild-type and mutants (Tukey’s test, ^∗^*P* < 0.05).

The total lipid extracted from leaf and separated on the TLC plate, showed spots of MGDG and DGDG, which were identified according to the result of Wang and Benning (2011). The MGDG spots of the Xidao #1 were comparatively bigger than the MGDG spots of the mutants (Figure 5A). The FAME content of MGDG was found significantly reduced by 12.8 and 10.5% in *osmgd2-1* and *osmgd2-2*, respectively. Similarly, The FAME content of DGDG was also reduced by 10.0 and 9.0% in the two mutants, respectively (Figure 5B).

#### Alteration of Viscosity of Rice Flour

To see whether lipid content change has an impact on the viscosity of milled rice, RVA was performed for milled rice flour of both mutants and their wild-type parent. The results showed significant differences in the setback and breakdown viscosity, i.e., the average setback viscosity was significantly increased (11.6 and 10.3%) while the breakdown viscosity was significantly reduced (21.2 and 18.6%) in the *osmgd2-1* and *osmgd2-2* mutants, respectively (Table 1). However, other parameters such as final viscosity and peak time did not differ significantly in mutants as compared to those in the wild-type.

**Table 1:** Rapid viscosity analysis (RVA) of milled rice of wild-type cultivar Xidao #1 and its two mutants *osmgd2-1* and *osmgd2-2.*

	Peak viscosity (RVU)	Trough (RVU)	Breakdown (RVU)	Final viscosity (RVU)	Setback viscosity (RVU)	Peak time (°C)
wild-type	152.6 ± 5.5	124.5 ± 1.2	35.2 ± 2.6	187.7 ± 4.7	35.1 ± 0.8	6.7 ± 0.2
*osmgd2-1*	149.4 ± 6.6	121.6 ± 3.6	27.8 ± 3.1	188.5 ± 7.4	39.2 ± 1.4	6.7 ± 0.1
*osmgd2-2*	164.6 ± 11.8	135.9 ± 15.8	28.6 ± 5.1	203.3 ± 12.7	38.7 ± 0.9	6.7 ± 0.2


### Impact of *osmgd2* Mutations on Vegetative and Yield Related Traits

#### *osmgd2* Mutants Had Lowered Photosynthetic Parameters

**FIGURE 6:**
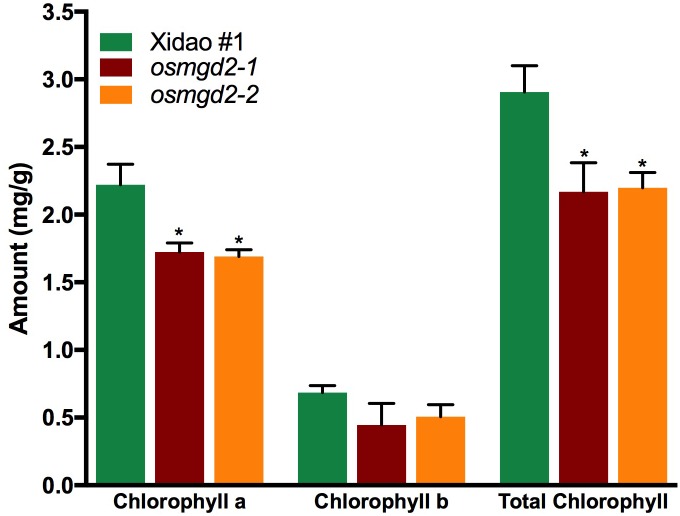
Chlorophyll content in leaf of wild-type cultivar Xidao #1 and its two mutants *osmgd2-1 and osmgd2-2*. All values represent means ± standard deviations of three biological replicates. Asterisk represents statistically significant differences between wild-type and mutants (Tukey’s test, ^∗^*P* < 0.05).

**FIGURE 7:**
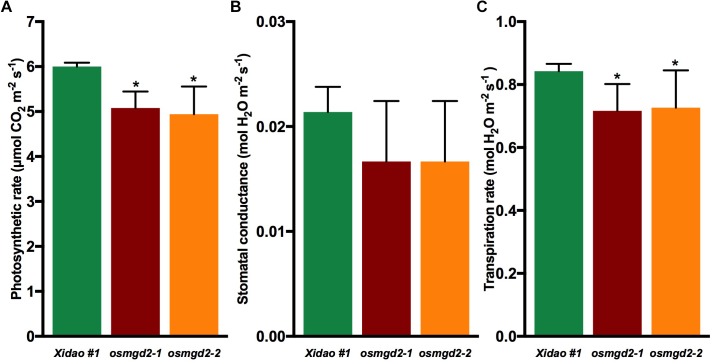
Photosynthesis parameters in wild-type cultivar Xidao #1 and its two mutants *osmgd2-1 and osmgd2-2.* **(A)** Photosynthetic rate, **(B)** Stomatal conductance and **(C)** Transpiration rate. All values represent means (standard deviations of three biological repeats. Asterisk represents statistically significant differences between wild-type and mutants (Tukey’s test, ^∗^*P* < 0.05).

The chloroplast localization of OsMGD2 and its substantial expression in leaf suggested that MGDG produced by OsMGD2 must play a role in leaf development and photosynthesis. Therefore, we studied the difference in chlorophyll content and photosynthetic rate between wild-type and the mutants. The chlorophyll **a** content was significantly reduced in the two mutants by 22.3 and 23.8%, respectively, while the reduction in chlorophyll **b** was insignificant (Figure 6). The photosynthetic rate in the two mutants reduced by 15.3 and 17.6%, while the transpiration rate was reduced by 15.3 and 13.7%. However, no notable difference was noted in the stomatal conductance (Figure 7).

#### *osmgd2* Mutants Had Lower Harvest Yield

**FIGURE 8:**
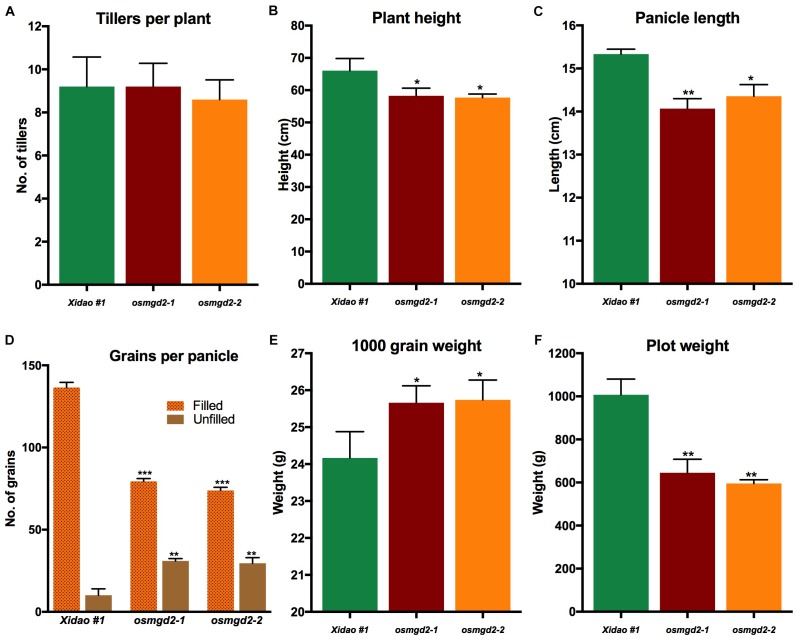
Agronomic and yield related traits of wild-type cultivar Xidao #1 and its two mutants *osmgd2-1 and osmgd2-2.* **(A)** number of tillers per plant, **(B)** plant height, **(C)** panicle length, **(D)** grains (filled and unfilled) per panicle, **(E)** 1000 grain weight and **(F)** plot yield. All values represent means ± standard deviations of 15 biological repeats for **A–D**; 9 biological repeats for **E**; and 3 biological repeats for **F**. Asterisk represent statistically significant differences between wild-type and mutants (Tukey’s test, ^∗^*P* < 0.05, ^∗∗^*P* < 0.01, ^∗∗∗^*P* < 0.001).

**FIGURE 9:**
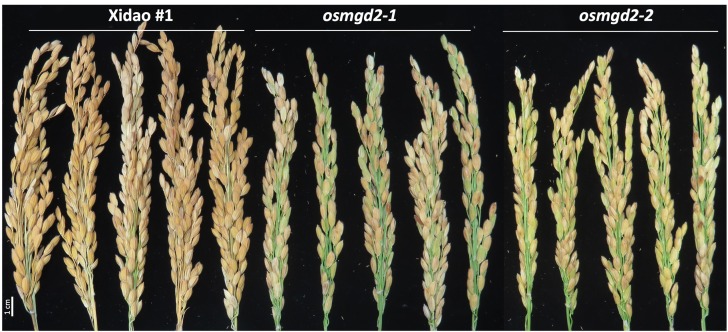
Panicles in wild-type cultivar Xidao #1 and its mutant *osmgd2-1* and *osmgd2-1.*

At the vegetative stage, no notable differences were observed between the wild-type and its two mutants and at maturity there was no significant difference in the average number of tillers per plants (Figure 8A). However, significant difference were observed for other traits at maturity (Figure 8). The plant height of two mutants was reduced by 11.8 and 12.6% as compared to the wild-type, and the panicle length was shortened by 8.2 and 6.4%, respectively (Figure 8C, 9). The total number of grains per panicle in two mutants was lessened by 41.8 and 45.9%, and the number of unfilled grains per panicle was increased in the two mutants by 205.9 and 192.1%, respectively (Figure 8D). However, the 1000 grain weight in the two mutants increased by 6.2 and 6.5% (Figure 8E). The plot weight was decreased from 1007.4 g in the wild-type to 644.9 and 595.8 g in its two mutants, respectively (Figure 8F).

## Discussion

### *OsMGD2* Is Expressed in Anther, Seed and Leaf

MGDG synthases in *Arabidopsis* have been classified into type-A and type-B on the basis of the length of the N-terminal portion, type A exhibiting a longer (∼100 aa) and type-B exhibiting a shorter (∼40 aa) N-terminal peptide, respectively (Awai et al., 2001; Kobayashi et al., 2004). In *Arabidopsis*, the majority of MGDG required for thylakoid biogenesis in leaf chloroplast is synthesized by the type-A MGDG synthase (AtMGD1*)*, while type-B MGDG synthases (AtMGD2 and AtMGD3) are active specifically in flowers and roots, as well as under stress conditions (Kobayashi et al., 2004, 2007). AtMGD2 and AtMGD3 have only a limited role in thylakoid membrane biogenesis but under phosphate (Pi)-deficient conditions, type-B MGDG synthase supply MGDG required for DGDG synthesis, which is involved in maintaining cellular homeostasis by substituting for phosphoglycerolipids (Kobayashi et al., 2009). Our phylogenetic analysis based on protein sequence also showed similarity with the phylogenetic relationship constructed on the basis of DNA sequences (Awai et al., 2001; Yuzawa et al., 2012), showing *AtMGD1* in the same clade with *OsMGD1* and *ZmMGD1*, while *AtMGD2* is in the same clade as *OsMGD2, OsMGD3, ZmMGD2*, and *ZmMGD3*. This relationship among MGDG synthases is also been supported by the motif analysis, which shows 11 conserved regions among AtMGD1, OsMGD1, and ZmMGD1, while there are only 10 conserved regions between MGD2 and MGD3 proteins of *Arabidopsis*, rice and maize. The common motif shared between the N-terminal peptides of AtMGD1, OsMGD2 and ZmMGD2 indicates that OsMGD2 and ZmMGD2 have longer N-terminal peptide similar to type-A MGDG synthase in *Arabidopsis*.

Comparative tissue specific expression study showed *MGD1* as the dominant gene in leaf in both *Arabidopsis* and rice, while in maize, expression of both *ZmMGD1* and *ZmMGD2* were predominant in leaf. We also noticed substantial expression of genes encoding MGDG synthase in floral tissues, particularly in anther and seeds, even higher than the expression in leaf, suggesting the importance of MGDG in flower and seed development. The biosynthesis and importance of MGDG in floral tissues have been highlighted in many green plants, including *Arabidopsis*, petunia and lily (Nakamura et al., 2003, 2009; Nakamura, 2015), particularly in the late stages of flower development (Nakamura et al., 2014). Plastids in non-photosynthetic tissues such as chromoplast and amyloplast have also been found to contain MGDG (Kalisch et al., 2016; Qin et al., 2019). Our gene expression analysis infers that the synthesis of MGDG in anther and seed is mainly contributed by *AtMGD3, OsMGD2*, and *ZmMGD2* in *Arabidopsis*, rice and maize, respectively. *AtMGD2*, which is the orthologous of *OsMGD2* and *ZmMGD2*, was found the least expressive in *Arabidopsis* in all tissues, while the expression of *OsMGD3* and *ZmMGD3* was found negligible in rice and maize, respectively. These expression profiles indicate that genes encoding MGDG in rice and maize are more conserved.

In rice, we noticed higher expression of *OsMGD2* in the anther and endosperm of mature seeds, implying the functions of MGDG synthases in non-photosynthetic tissues. The termination of protein translation before the functional domains suggests that the mutants carry a loss of functional OsMGD2. This mutation may have led to nonsense-mediated mRNA decay in both anther and leaf, thereby decreasing the *OsMGD2* mRNA levels as reported in other species (Gong et al., 2007). We also observed that the decrease in transcript level of *OsMGD2* was not compensated by the increase in expression of its homologous genes in both anther and leaf. This indicates that *OsMGD2* is pivotal for synthesizing MGDG in floral as well as leaf tissues of rice.

The activation of MGDG synthase has been shown to be regulated by many other intrinsic and extrinsic factors. The galactolipid content was found to be greatly increased in response to light and exogenous cytokinin treatment in cucumber (Yamaryo et al., 2003) and *Arabidopsis* (Kobayashi et al., 2014). Similarly, redox has also been found to reversibly regulate the enzymatic activity of MGDG synthesis in cucumber (Yamaryo et al., 2006) and sesame (Shimojima et al., 2013). Other factors such as anionic phospholipids, phosphatidic acid and phosphatidylglycerol have also been reported to activate plant MGDG synthases (Dubots et al., 2010; Shimojima et al., 2013). Similarly, various transcriptional factors such as HY5 and GLK are also involved in the regulation of MGDG synthase (Kobayashi et al., 2014). Likewise, GUN1, a central regulator of plastid signaling, might also play a pivotal role in regulation of galactolipid synthesizing genes via GUN1-mediated plastid signaling (Koussevitzky et al., 2007; Kobayashi and Wada, 2016). Therefore, these complex regulatory mechanisms might be involved in differential activity of MGDG synthase in different tissues.

### Chloroplast Localization of OsMGD2 Signifies Its Involvement in Photosynthesis

Chloroplast is the site of oxygenic photosynthesis in cyanobacteria, algae and higher plants. The thylakoid membrane of chloroplast contains high abundance of MGDG and DGDG that are critical for the light reactions of photosynthesis. The chloroplast localization of OsMGD2, which is similar to AtMGD1, AtMGD2 and AtMGD3 (Awai et al., 2001), indicates the similarity in synthesis and function of MGDG contributed by OsMGD2. The inner and outer membrane of chloroplast envelope has been suggested as the site for synthesis of MGDG, which are then transported inside to synthesize the thylakoid membrane (Dörmann and Benning, 2002). AtMGD1 has been proposed to be associated to the inner envelope of the chloroplast, whereas AtMGD2 and AtMGD3 are targeted to outer envelopes and are considered less important for thylakoid membrane biogenesis (Benning and Ohta, 2005). AtMGD1 together with AtDGD1 was found essential for synthesis of bulk of galactolipids in *Arabidopsis* (Jarvis et al., 2000; Kobayashi et al., 2007) and are found to be highly expressed in tissues requiring rapid chloroplast development, such as young leaves (Benning and Ohta, 2005).

*osmgd2* mutants were characterized by reduction in MGDG and DGDG content. MGDG is the substrate for DGDG synthesis, therefore, the reduction in MGDG could have resulted in the decrease of DGDG content in leaves. The reduction in galactolipids would have consequently caused the depletion of the chlorophyll **a** content in *osmgd2* leaves, similar to previous reports in other plants (Kobayashi et al., 2007, 2014). Chlorophyll **a** is the main photosynthetic pigment directly involved in transfer of the electron during the light transformation reaction, while chlorophyll **b** acts as light absorbing pigment (Yahia et al., 2019). The substantial expression of *OsMGD2* in leaves, its extended N-terminal region and decrease in galactolipid content in mutant leaves provide strong evidences for the contribution of OsMGD2 in synthesizing MGDG required for chloroplast biogenesis, contributing to photosynthesis in leaves. Photosynthesis also occurs in the outer green pericarp of seeds, which provides energy to support the actively metabolizing seed inside (Tschiersch et al., 2011). The interior of developing seeds is typically hypoxic and requires constant supply of O_2_ through seed photosynthesis, thereby maintaining endogenous O_2_ balance and limiting fermentation (Rolletschek et al., 2004; Tschiersch et al., 2011). This signifies the role of MGDG not only in the leaves, but also in the grains for conducting photosynthesis required for seed development.

### Mutation of *OsMGD2* Affects Seed Quality and Agronomic Performance

The lipid is a minor component in rice grain, which constitutes less than 1% of the milled rice (Fujino, 1978; Choudhury and Juliano, 1980). However, it could affect the functionality and metabolism of starch molecules (Zhou et al., 2003; Tong and Bao, 2019). The majority of lipids in rice endosperm are phospholipids and its derivatives, which are complexed with the starch molecules forming the amylose-lipid complex, thus have quality and nutritional significance in rice (Liu et al., 2013). It has been shown that rice with better eating quality has higher starch lipid and linoleic acid contents (Yoon et al., 2012).The reduction of linoleic acid content in *osmgd2* milled rice is hence consistent with the inferior characteristics of RVA profile, i.e., with reduced breakdown and increased setback values. This highlights the importance of *OsMGD2* as one of the principal genes synthesizing galactolipids in rice endosperm and has important functions in development and productivity of the grain.

Linoleic acid has been reported as a dominant fatty acid in maize, constituting 67% of the galactolipid in endosperm (Myers et al., 2011; Yoon et al., 2012). The decrease in MGDG synthesis in maize was found to result in abnormal development of endosperm with highly vacuolated cells and decreased number of starch grains, while complete loss of function of MGD1 in a null mutant (*O5*) resulted in kernel lethality (Myers et al., 2011). Lipid content has also been correlated with the gelatinization and pasting properties of starch, indirectly relating to the eating quality in sensory rice evaluation (BeMiller, 2019). The alteration in the setback and breakdown viscosity in the RVA profiling of *osmgd2* mutants, indicate the association between MGDG and eating quality in rice. These changes are caused possibly due to the disruption in the starch granule structure caused by the decrease in the fatty acid content in the milled rice (BeMiller, 2019; Tong and Bao, 2019). The parameters analyzed in RVA provide discrete values related with the pasting properties of starch, as well as the texture and quality of milled rice (Cozzolino, 2016). Setback refers to the difference between final and peak viscosity, strongly predicting the final texture and indicating the firmness of cooked rice, and therefore has been used as a selection tool in rice breeding programs for rice eating quality (Balindong et al., 2018).

The loss of function of OsMGD2 had a negative impact on the overall development and productivity of the rice. The reduction in MGDG has detrimental effect on the photosynthesis and overall lipid synthesis in the seeds. Consequently, the *osmgd2* mutants had shortened plant height, reduced panicle length, and fewer number of filled grains per panicles. The increase in number of unfilled grains per panicle highlights the importance of MGDG in the development and fertilization in floral organs. Galactolipids have been found to have important roles in flower development, particularly DGDG which has higher activity and synthesis in pistil and elongated pollen tubes of Lily and petunia (Nakamura et al., 2003, 2009). In another report, DGDG was found to accumulate in the plasma membrane of elongated pollen tubes, and inhibition in MGDG synthase impairs pollen growth in *Arabidopsis* (Botté et al., 2011). Therefore, our findings further indicate galactolipids play important roles in the development of floral organs and fertilization, as evidenced by the reduced seed-set rate observed in the *osmgd2* mutants.

## Conclusion

In this study, three genes encoding MGDG synthase in rice were identified and their gene structures, motifs and domains in proteins were studied. Mutational analysis showed *OsMGD2* as an important MGDG synthesizing gene in grain and anther, as well as in leaf of rice. The loss of function of OsMGD2 leads to reduction in plant productivity and linoleic acid content in milled rice, thus altered starch pasting properties. These results hence signify the non-photosynthetic functions of galactolipids in rice grain development, which also indicate the diversification of functions of genes encoding MGDG synthase between *Arabidopsis* and rice.

## Data Availability

All datasets generated for this study are included in the manuscript and/or the Supplementary Files.

## Author Contributions

RB conducted the research work and prepared the manuscript. JZ helped in tissue culture work for generating mutants as well as collecting agronomic data in the field. NH helped technically to analyze lipids using the GC-FID. QS is the supervisor of the research work and also the corresponding author.

## Conflict of Interest Statement

The authors declare that the research was conducted in the absence of any commercial or financial relationships that could be construed as a potential conflict of interest.

## Footnotes

1 http://www.gramene.org/

2 http://www.genome.jp/kegg/kegg2.html

3 https://www.plantcyc.org/

4 http://www.arabidopsis.org/

5 http://www.ncbi.nlm.nih.gov

6 http://rice.plantbiology.msu.edu/

7 http://gsds.cbi.pku.edu.cn/

8 https://www.ebi.ac.uk/Tools/hmmer/

9 http://meme-suite.org/tools/meme

10 http://bar.utoronto.ca/

11 http://crispr.hzau.edu.cn/CRISPR/

12 http://web.expasy.org/translate/
