# Supplementary material for: Characterization and Mutational Analysis of a Monogalactosyldiacylglycerol Synthase Gene OsMGD2 in Rice
Source: Front Plant Sci. 2019 Aug 2;10:992. doi: 10.3389/fpls.2019.00992 (PMC6688468; doi:10.3389/fpls.2019.00992)
Supplement: Supplementary file 1 [file Table_1.docx]

Supplementary Figures and Tables


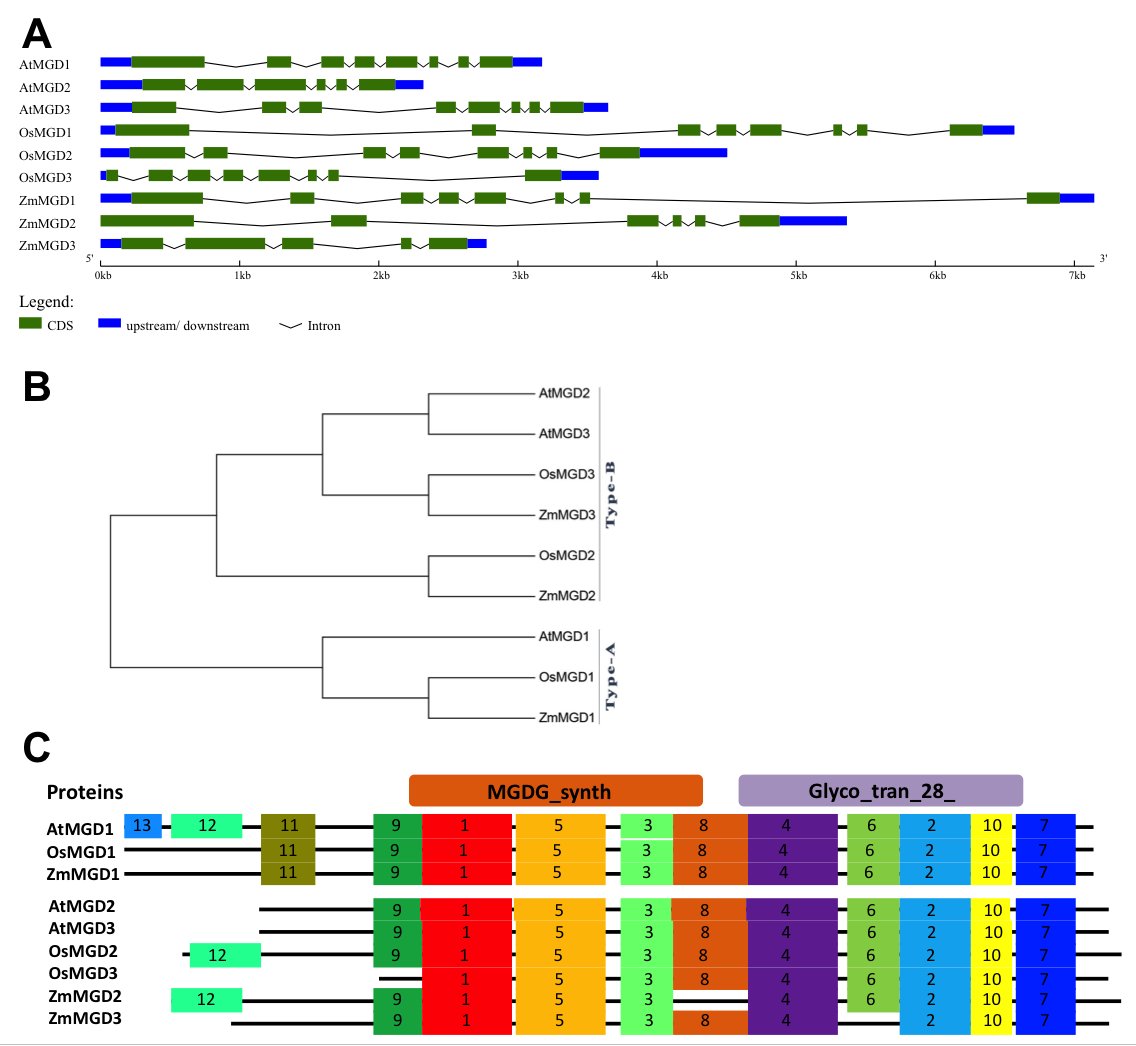


**Supplementary Figure S1**. ***In silico* Analysis:** **A**. Intron-exon structure of genes encoding MGDG synthase in *Arabidopsis*, rice and maize. **B.** Phylogenetic relationship between *Arabidopsis*, rice and maize MGDG synthase proteins. **C.** Distribution of conserved motifs and domains in MGDG synthase proteins of *Arabidopsis*, rice and maize*.* Motifs are represented by colored boxes with numbers, the two common domains are represented by orange (MGDG_synth) and purple (Glyco_tran_28_C) colored boxes at the top. The position of domains is based on the *AtMGD1*.

**Supplementary FigureS2.** Orthologous of genes encoding MGDG synthase in rice, *Arabidopsis* and maize and their expression in leaf, seed, anther and pistil. Relative expression values for rice was obtained from the RGAP database, while absolute expression values for *Arabidopsis* and maize were obtained from the BAR (The Bio-Analytic Resource for Plant Biology) database.

Supplementary Table S1. List and sequences of oligos and primers

| Sn | Primers | Sequence (5'-3') |
| --- | --- | --- |
| 1 | M2-T | GGCAGAGGATGAGGCGCAAGTTC |
| 2 | M2-B | AAACGAACTTGCGCCTCATCCTC |
| 3 | M2-F | TCAAGTTATGGCATTTTCCGTCT |
| 4 | M2-R | GCACCCTTGAAGAATGCTTGT |
| 5 | seq-F | GCCCATTACGCAATTGGACG |
| 7 | M2-cD-F | ATGGCGGCGTCGTCGTCGTCG |
| 8 | M2-cD-R | CATAAGTTGTATAGGTGTGG |
| 9 | M2-HR-F | tttggagaggacacgctcgagATGGCGGCGTCGTCGTCGTCG |
| 10 | M2-HR-R | cctcgcccttgctcaccatggCATAAGTTGTATAGGTGTGG |
| 11 | M1-RT-F | TCGTGGCCTGCCCATTATAC |
| 12 | M1-RT-R | ACCTAGGGCCAAACCAATCG |
| 13 | M2-RT-F | TCGCCGAAGCCTTGATTAGA |
| 14 | M2-RT-R | TTGGAGAAGACACCAGCACC |
| 15 | M3-RT-F | TGGTACAATTGCAGAGGCCC |
| 16 | M3-RT-R | CTCGGGTCCTTGGAGAACAC |
| 17 | Actin-F | GAACTGGTATGGTCAAGGCTG |
| 18 | Actin-R | ACACGGAGCTCGTTGTAGAAG |
| 19 | Hyg-F | AGAAGAAGATGTTGGCGACCT |
| 20 | Hyg-R | GTCCTGCGGGTAAATAGCT |

Supplementary Table S2. Predicted features of MGDG synthase genes in rice

| Gene | Gene ID | | Location | Protein Length | MW | pI |
| --- | --- | --- | --- | --- | --- | --- |
|  | RAP | MSU |  |  |  |  |
| *OsMGD1* | Os09g0423600 | LOC_Os09g25580 | 9:15355640 - 15349072 | 536 | 56924.9 | 8.5882 |
| *OsMGD2* | Os02g0802700 | LOC_Os02g55910 | 2:34223117 - 34227448 | 509 | 56217.6 | 7.9298 |
| *OsMGD3* | Os08g0299400 | LOC_Os08g20420 | 8:12266091 - 12269671 | 395 | 44644.6 | 9.2602 |
